# Smelling the Diagnosis: The Electronic Nose as Diagnostic Tool in Inflammatory Arthritis. A Case-Reference Study

**DOI:** 10.1371/journal.pone.0151715

**Published:** 2016-03-16

**Authors:** Marjolein P. Brekelmans, Niki Fens, Paul Brinkman, Lieuwe D. Bos, Peter J. Sterk, Paul P. Tak, Daniëlle M. Gerlag

**Affiliations:** 1 Division of Clinical Immunology and Rheumatology, Academic Medical Centre, University of Amsterdam, Amsterdam, the Netherlands; 2 Department of Respiratory Medicine, Academic Medical Centre, University of Amsterdam, Amsterdam, the Netherlands; 3 Department of Intensive Care Medicine, Academic Medical Centre, University of Amsterdam, Amsterdam, the Netherlands; Queen Mary University of London, UNITED KINGDOM

## Abstract

**Objective:**

To investigate whether exhaled breath analysis using an electronic nose can identify differences between inflammatory joint diseases and healthy controls.

**Methods:**

In a cross-sectional study, the exhaled breath of 21 rheumatoid arthritis (RA) and 18 psoriatic arthritis (PsA) patients with active disease was compared to 21 healthy controls using an electronic nose (Cyranose 320; Smiths Detection, Pasadena, CA, USA). Breathprints were analyzed with principal component analysis, discriminant analysis, and area under curve (AUC) of receiver operating characteristics (ROC) curves. Volatile organic compounds (VOCs) were identified by gas chromatography and mass spectrometry (GC-MS), and relationships between breathprints and markers of disease activity were explored.

**Results:**

Breathprints of RA patients could be distinguished from controls with an accuracy of 71% (AUC 0.75, 95% CI 0.60–0.90, sensitivity 76%, specificity 67%). Breathprints from PsA patients were separated from controls with 69% accuracy (AUC 0.77, 95% CI 0.61–0.92, sensitivity 72%, specificity 71%). Distinction between exhaled breath of RA and PsA patients exhibited an accuracy of 69% (AUC 0.72, 95% CI 0.55–0.89, sensitivity 71%, specificity 72%). There was a positive correlation in RA patients of exhaled breathprints with disease activity score (DAS28) and number of painful joints. GC-MS identified seven key VOCs that significantly differed between the groups.

**Conclusions:**

Exhaled breath analysis by an electronic nose may play a role in differential diagnosis of inflammatory joint diseases. Data from this study warrant external validation.

## Introduction

Rheumatoid arthritis (RA) is a systemic immune-mediated inflammatory disease predominantly affecting the joints. The prevalence of RA is about 1% worldwide, increases with age and the average age at onset is 30–50 years[1]. The aetiology of RA is not yet elucidated although genetic factors and lifestyle-related factors, such as smoking and obesity, have been implied in the pathogenesis[2–4]. The diagnosis is based on clinical signs and symptoms, supplemented with laboratory and radiographic tests. Despite the advances in the development of novel diagnostic tools, such as MRI and ultrasound[5, 6], diagnosing RA can be challenging during the earliest phases of the disease due to similarities with other inflammatory joint diseases[7] and its variable presentation. Early diagnosis is important to prevent therapeutic delay, because of the risk of irreversible damage and destruction of cartilage and bone with disability and decreased quality of life as a result[8, 9]. Distinguishing RA from other inflammatory diseases is of importance, because of differences in prognosis and treatment regimens[10, 11]. Therefore, improvement of early diagnosis of the different inflammatory joint diseases using novel tools is warranted.

Exhaled breath comprises gases and many hundreds of volatile organic compounds (VOCs, metabolites), which are derived from multiple metabolic and inflammatory processes taking place in the human body[12]. Considering the fact that inflammatory joint diseases are systemic diseases, it is likely that VOCs representing inflammation and activity of these diseases can be found in the lungs and in the exhaled breath[13], as has been shown in asthma and chronic obstructive pulmonary disease (COPD)[14], infectious diseases[15] and different forms of cancer[16–18]. Gas chromatography and mass spectrometry (GC-MS) can be used to detect these VOCs and their concentrations on an individual basis[12, 19, 20]. Electronic noses (eNoses) are devices that allow high-throughput analysis of mixtures of gases representing an innovative method to measure the complete spectrum of VOCs as a composite fingerprint[21, 22]. Because of the non-invasive character of the exhaled breath collection and the possibility to analyse the complete VOC spectrum, eNoses could have potential as a diagnostic tool[13, 22–24].

We hypothesized that an eNose may be used to discriminate exhaled breath of patients with RA from patients with psoriatic arthritis (PsA) and healthy volunteers, and explored this in a case-control study. In addition we investigated whether the exhaled breath prints are associated with systemic inflammatory and disease activity markers. GC-MS analysis was performed to identify possible disease specific VOCs in the patients studied.

## Materials and Methods

Sixty subjects were included in the study. All subjects were non-smokers and aged 18 to 77 years. The study population comprised 3 different groups: (1) RA patients (n = 21), (2) PsA patients (n = 18), and (3) control subjects (n = 21). Patients were recruited from the outpatient clinic of the department of Clinical Immunology and Rheumatology of the Academic Medical Center and allied hospitals. Healthy controls were recruited by means of advertisement in the hospital and the medical faculty of the University of Amsterdam.

The 21 RA patients fulfilled the ACR criteria[25] and had active disease defined by a disease activity score of 28 joints (DAS28) ≥ 3.2[26]. The 18 PsA patients fulfilled the CASPAR criteria[27] and had active disease defined by a DAS28 ≥ 3.2. The control group consisted of 21 healthy subjects who were IgM rheumatoid factor (IgM RF) and anti- citrullinated peptide antibody (ACPA) negative, as measured by the anti-cyclic citrullinated peptide CCP) test.

Exclusion criteria were history of cardiovascular disease, diabetes mellitus, systemic inflammatory disease other than RA or PsA, active pulmonary disease, current or past malignancy, unstable hyper- or hypothyroid function, renal or liver insufficiency and pregnancy. Furthermore, the use of inhalation medication, oral or systemic corticosteroids > 10 mg a day, intramuscular or intraarticular corticosteroids in the last 4 weeks, and treatment with biologicals 1 to 12 months before the study (etanercept < 1 month, or adalimumab, infliximab, tocilizumab or abatacept < 3 months, or rituximab < 12 months) were exclusion criteria.

The study was approved by the Medical Ethics Committee of the Academic Medical Centre and all subjects gave written informed consent prior to study participation.

### eNose procedure

The study had a cross-sectional case-reference design with a single visit to the outpatient clinic. Exhaled breath collection was performed by a validated procedure as described before[28, 29]. Briefly, patients breathed normally with a clip on their nose through a mouthpiece for 5 minutes. The mouthpiece was attached to a three-way non re-breathing valve, with on the inspiratory side a VOC-filter (A2, North Safety, Middelburg, the Netherlands) and on the expiratory side a silica filter to dry the exhaled breath. After a maximal inspiration, a single vital capacity volume was exhaled by the patient into a 10L Tedlar bag (SKC Inc., Eighty Four, PA, USA) connected to the silica reservoir at the expiratory port. Within 30 minutes after exhaled breath collection, the patient’s Tedlar bag was connected to the eNose (Cyranose 320; Smiths Detection, Pasadena, CA, USA), followed by bag sampling for the duration of 1 minute. In parallel, a second Tedlar bag containing VOC-filtered room air for comparison (background air sample) was sampled[29]. The Cyranose settings were Baseline purge: 30 sec. [pump setting: medium], Sample draw 1: 60 sec. [pump: medium], Snout removal: 0 sec., 1st sample gas purge: 0 sec., 1st air intake purge: 10 sec. [pump: high], 2nd sample gas purge: 200 sec. [pump: high], 2nd air intake purge: 0 sec., Substrate heater: 42.0 degrees Celsius. The measurements of the exhaled breath samples by the eNose were performed in duplicate.

To find potential differences in exhaled substances between the various groups, explorative analysis of the exhaled breath stored on Tenax GR filled adsorption tubes (TD100; Markes, Cincinnati) by gas chromatography and mass spectrometry (GC-MS), as described earlier[28, 30], was additionally done for 5 patients of each group. In short, adsorption tubes were thermally desorbed followed by split less injection onto the chromatographic column. Compounds were separated using capillary gas–chromatography with helium as a carrier gas (6890N GC, Agilent, Santa Clara, CA, USA) on a VF1–MS column. A quadrupole mass spectrometer (5975 MSD, Agilent, Santa Clara, CA, USA) was used for the detection of product ions. Peak–detection and alignment were performed using the Xcms–package for R[31]. Ion-fragments were grouped based on retention time and correlation coefficients categories that represent VOCs. Fragmented ions were tentatively identified based on NIST–library matching. When this procedure did not result in identification, the compound was called “unknown”.

### Statistical analysis

SPSS (version 21) and Graphpad Prism (version 5.01) were used for analysis of acquired raw eNose data and R (v2.14, www.r-project.org) using the R-studio interface for GC-MS data. Differences between groups were analyzed using chi-square for categorical variables and with one-way ANOVA with post-hoc student t-tests for continuous variables. P-values below 0.05 were considered statistically significant. During every measurement with the eNose the ∆R/R for each of the 32 sensors was saved. These raw eNose data were reduced by principal component analysis (PCA) from 32 sensors to four principal components (PC). Significantly different PCs were used as independent variables for linear canonical discriminant analysis[24, 32]. We have recently compared various statistical techniques for dimension-reduction and classification of disease with electronic nose signals. In this comparative analysis that included all datasets that performed external validation, principal component analysis with linear discriminant was found to be equal to, or possibly slightly better, than non-linear classification tools such as support vector machines and neural networks[33].

The leave-one-out cross-validated accuracy was reported (internal validity). Receiver operating characteristic (ROC) curves were constructed based on the discriminant functions and the area under the curve (AUC) with 95% confidence intervals (95% CI) were reported. The relationship between the markers of active disease (ESR, DAS28, and number of painful and swollen joints) and the principal components were analyzed with Pearson’s correlation coefficient (r). VOCs detected by GC-MS were compared between the three groups using one-way ANOVA.

## Results

Baseline characteristics of the 60 subjects are summarized in Table 1. All patients were non-smokers. Patients with RA and PsA were significantly older than controls (p = 0.001 and p = 0.01 respectively), but there was no significant difference between the age of RA and PsA patients (p = 0.461). Patients with RA used different medication than PsA patients (p = 0.037) and controls (P < 0.001), see frequencies in Table 1. There were no other significant differences in subject characteristics between the three groups.

**Table 1:** Characteristics of the study population.

Subject characteristics	RA patients	PsA patients	Controls
n	n = 21	n = 18	n = 21
Age (years) in mean (SD)	53.5 (14.8)	50.1 (12.9)	39.1 (12.1)
Sex (M/F)	5/16	7/11	4/17
Ethnicity (white/other)	16/5	15/3	17/4
Rheumatoid factor (positive/negative)	9/12	2/16	0/21
ACPA (positive/negative)	16/5	0/18	0/21
Alcohol consumption (yes/no)	14/7	10/8	18/3
Medication use			
*No medication*	3	11	21
*Methotrexate*	10	5	0
*Hydroxychloroquine*	2	1	0
*Sulfasalazine*	0	0	0
*Leflunomide*	0	0	0
*Prednisone*	2	0	0
*Combination ->*	4	1	0
Methotrexate + prednisone	2	0	0
Methotrexate + hydroxychloroquine	1	1	0
Leflunomide + sulfasalazine	1	0	0

*Definition of abbreviations*: RA = rheumatoid arthritis, PsA = psoriatic arthritis, ACPA = anti-citrullinated peptide antibodies

### RA patients versus controls

**Fig 1:**
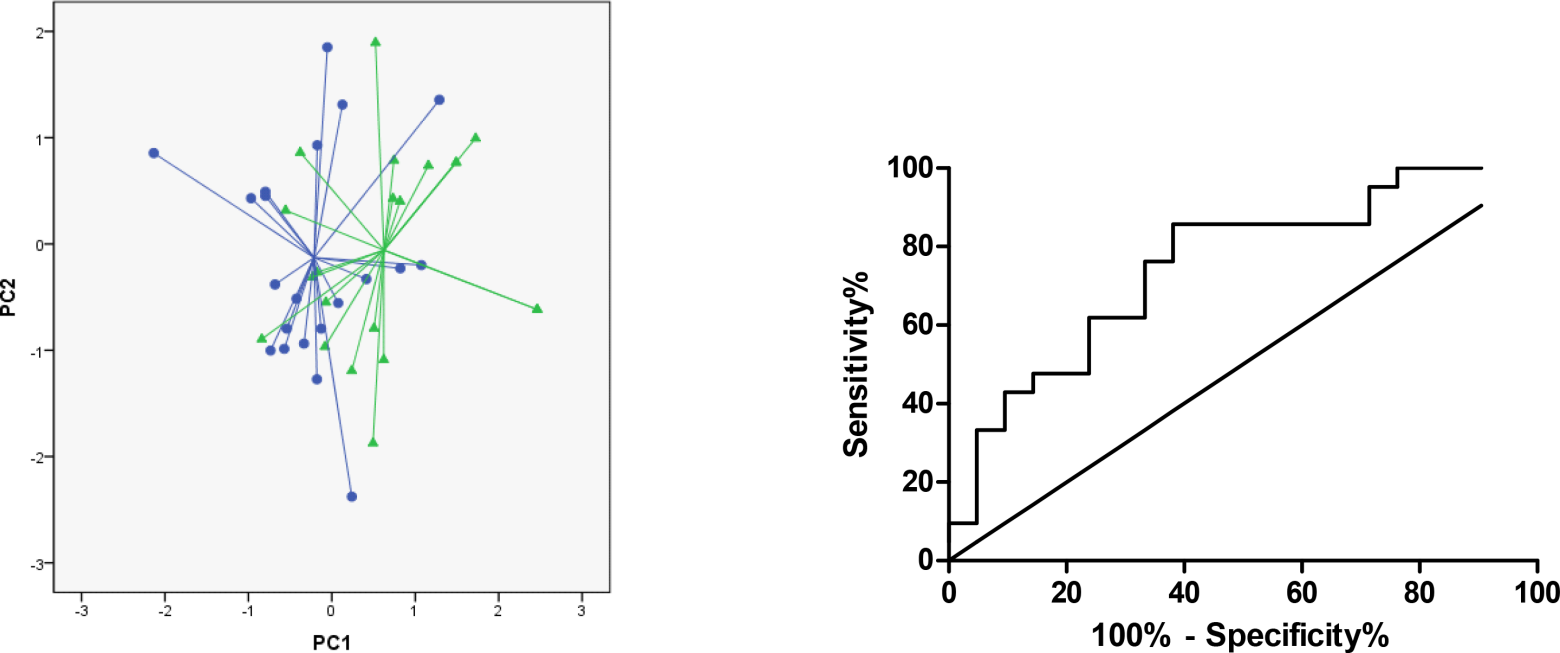
Comparison of breathprints of patients with RA (*circles*) versus controls (*triangles*). (A) Two-dimensional principal component plot showing the discrimination of breathprints of patients with RA and controls. Accuracy of 71% (P = 0.003). (B). Receiver operator characteristics (ROC) curve for RA vs controls with line of identity of the breathprint discriminant function (representing PC 1). AUC reached 0.75.

Principal component and canonical discriminant analysis showed that based on PC1 patients with RA could be distinguished from healthy controls with an accuracy of 71% (p = 0.003) (Fig 1A). The area under curve (AUC) was 0.75 (95% CI 0.60–0.90) (Fig 1B), with optimal sensitivity of 76% (95% CI 53–92%) and specificity of 67% (95% CI 43–85%), LR+ 2.3 and LR- 0.36 (Table 2).

**Table 2:** Cross-validation values for the discrimination between patients with RA, PsA and controls.

Set	Acc (%)	P-value	AUC	95% CI	Sens (%, 95% CI)	Spec (%, 95% CI)	LR+	LR-
RA vs. controls	71	0.003	0.75	0.60–0.90	76 (53–92)	67 (43–85)	2.3	0.36
PsA vs. controls	69	0.014	0.77	0.61–0.92	72 (47–90)	71 (48–89)	1.8	0.40
RA vs. PsA	69	0.026	0.72	0.55–0.89	71 (47–90)	72 (48–89)	2.5	0.40

*Definition of abbreviations*: Acc, accuracy; AUC, area under the curve; 95% CI, 95% confidence interval; Sens, sensitivity; Spec, specificity; LR+, positive likelihood ratio; LR-, negative likelihood ratio.

### PsA patients versus controls

**Fig 2:**
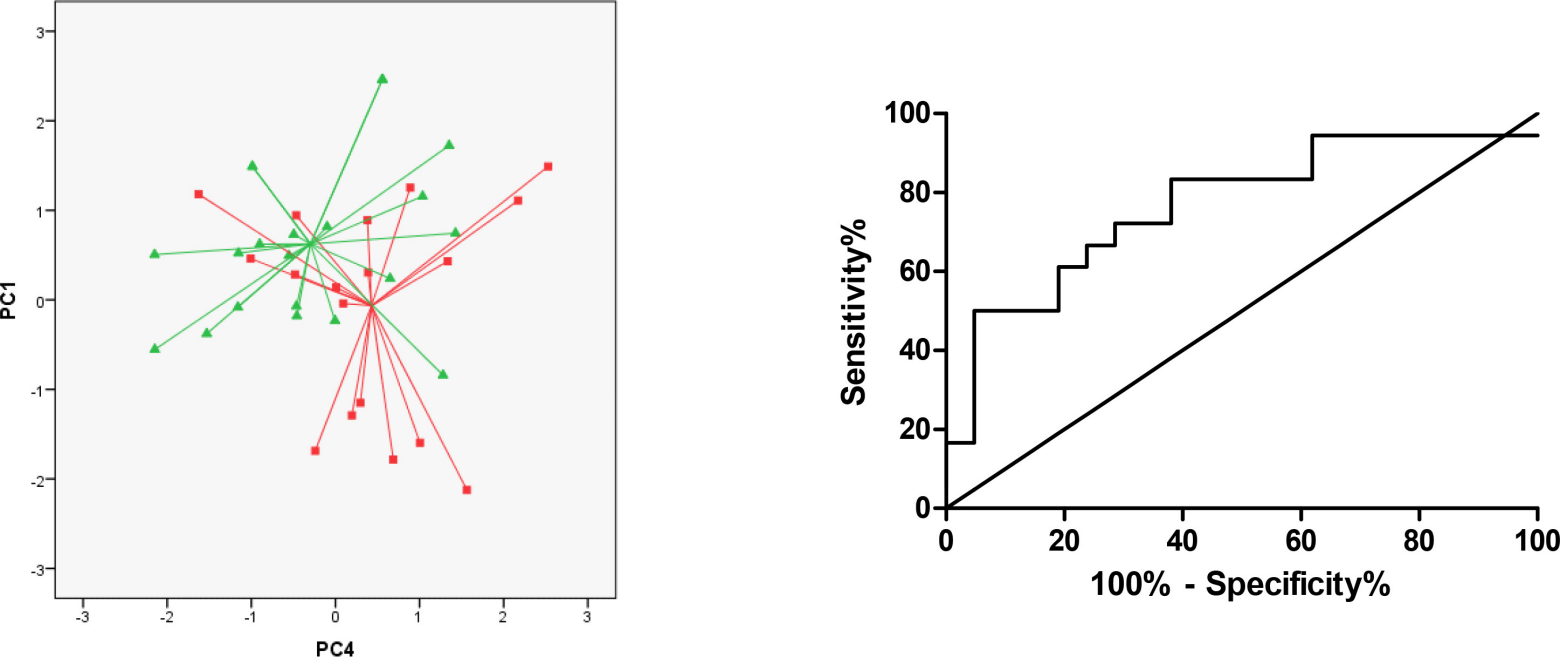
Comparison of breathprints of patients with PsA (*squares*) versus controls (*triangles*). (A) Two-dimensional principal component plot showing the discrimination of breathprints of patients with PsA and controls. Accuracy of 69% (P = 0.014). (B) Receiver operator characteristics (ROC) curves for PsA vs. controls with line of the breathprint discriminant function (representing PC 1 and 4). AUC was 0.77.

Using PC1 and 4, patients with PsA could be distinguished from healthy controls with a cross validated accuracy of 69% (p = 0.014) (Fig 2A). The AUC reached 0.77 (95% CI 0.61–0.92) (Fig 2B), with sensitivity 72% (95% CI 47–90%) and specificity 71% (95% CI 48–89%) and LR+ 2.5 and LR- 0.40 (Table 2).

### RA versus PsA patients

**Fig 3:**
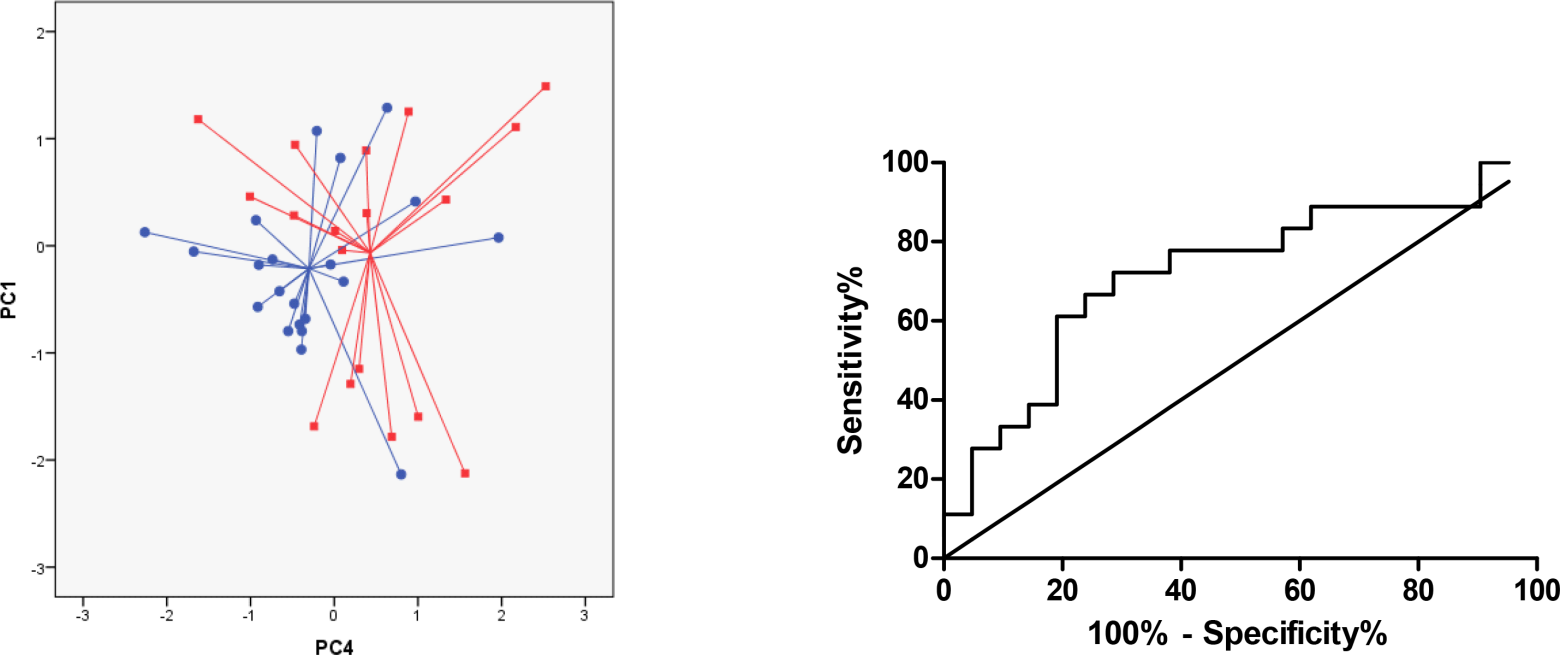
Comparison of breathprints of patients with RA (*circles*) versus PsA (*squares*). (A) Two-dimensional principal component plot showing the discrimination of breathprints of patients with RA and PsA. Accuracy of 69% (P = 0.026). (B) Receiver operator characteristics (ROC) curves for PsA vs. controls with line of the breathprint discriminant function (representing PC 4). AUC was 0.72.

Patients with RA could be distinguished from patients with PsA using breathprint PC 4. The accuracy for discrimination reached 69% (p = 0.026) (Fig 3A). The AUC of the ROC curve was 0.72 (95% CI 0.55–0.89) (Fig 3B) with optimal sensitivity 71% (95% CI 47–90%) and specificity 72% (95% CI 48–89%), LR+ 2.5 and LR- 0.40 (Table 2).

### Correlation analysis of inflammatory markers and eNose breathprints

In RA patients, there was a correlation between PC1 and number of painful joints (r = -0.47, p = 0.032), PC1 and DAS28 (r = -0.45, p = 0.041), PC3 en DAS 28 (r = -0.47, p = 0.034) and between PC4 and number of painful joints (r = -0.55, p = 0.010). There were no significant correlations between breathprints and ESR, and total number of swollen joints.

In PsA patients there were no significant correlations found between breathprints and markers of active disease.

### GC-MS analysis

Explorative GC-MS analysis showed seven VOCs (2-Propanol; 1-Propanol, 2,2-dimethyl; n-Hexanol; 2-Pentanone; Unknown 1; Unknown 2: Unknown 3) to significantly differ between the three groups. Four of the seven VOCs were found in lower concentration in the exhaled breath of patients with RA compared to control subjects, while three VOCs were higher in PsA patients than in controls (Table 3). Comparing RA with PsA, five VOCs were shown to be lower in RA patients than in PsA patients.

**Table 3:** VOCs (metabolites) found in significantly different concentrations in RA patients versus controls and RA versus PsA patients.

VOC	RA vs. control	PsA vs. controls	RA vs. PsA
2-Propanol	≈	↑	↓
1-Propanol, 2,2-dimethyl	↓	↑	↓
n-Hexanol	↓	≈	↓
2-Pentanone	↓	≈	↓
Unknown 1	≈	↑	≈
Unknown 2	↓	≈	↓
Unknown 3	≈	≈	↑

## Discussion

This study shows for the first time that the use of breath analysis by an electronic nose is able to discriminate patients with active RA from patients with active PsA and healthy controls with moderate accuracy. The exploratory GC-MS analysis confirmed these distinctions with a separation between RA and PsA patients and the control subjects. These findings show that the mixture of VOCs in the exhaled breath of patients with RA is different in comparison to patients with PsA and control subjects, which provides the rationale for future prospective studies to validate the value of the use of an eNose in the differential diagnosis of patients with early arthritis.

The eNose has been evaluated in different conditions, such as type II diabetes type, infectious diseases and cancer[17, 34, 35]. The use of an eNose in respiratory diseases showed that breathprints from patients with asthma, COPD and lung cancer can be distinguished from control subjects and from each other[28, 29, 36]. An eNose has not been tested before in patients with chronic inflammatory joint diseases, but measurement of H_2_O_2_ in the fluid phase of exhaled breath condensate has been reported earlier and has suggested a trend towards increased levels in RA patients compared to healthy controls[37]. The breathprints of patients with arthritis showed a distinction between RA, PsA and controls. The results suggest that the VOCs in the exhaled breath may function as diagnostic biomarkers for the discrimination between patients with different forms of arthritis and controls.

There are several methodological aspects that need further discussion. First, some baseline characteristics differed between the groups, such as age and medication usage. We cannot completely exclude the possibility that different processes related to age may have influenced the exhaled breath profiles. However, this appears less likely as earlier research showed no difference between the exhaled breath profiles between younger and older controls[28]. The use of medication could also be a confounding factor, although the statistically significant difference between the breathprints of PsA patients, most of whom did not use medication, and healthy controls suggest that this might not play a major role. Second, the method of breath collection and sampling is of great importance to obtain useful and reproducible results from the eNose analysis. We used a previously validated procedure to minimize the external influence on VOCs in the exhaled breath samples[28, 29]. Finally, because repeated measurements confirmed the differences found, it is unlikely that the findings of this study are attributable to chance or error.

The explorative GC-MS analysis showed the presence of seven VOCs that were significantly different between the groups. These VOCs are likely the result of the body’s response to inflammation, but may also be important in the disease processes of RA and PsA. Although RA and PsA share common mechanisms, such as a crucial role for TNF, there are evident differences in pathogenesis that are apparently associated with distinct metabolic processes[38, 39]. Other metabolomics studies in RA and PsA patients focusing on peripheral blood and urine samples showed different metabolites than our GC-MS analysis of VOCs in the exhaled breath[40, 41]. This difference might be a reflection of different underlying metabolic pathways leading to excretion by the kidney versus the lung.

In conclusion, a handheld, non-invasive, easy to use eNose is suggested to differentiate to some extent between the breathprints of patients with active RA, active PsA and healthy controls and could possibly provide an additional tool in the diagnosis of inflammatory joint diseases. The data presented warrant external validation of this tool by testing its diagnostic accuracy prospectively in a newly and large untreated group of patients with signs and symptoms of joint inflammation in various diseases.
